# Recent advances in vitamin D implications in chronic respiratory diseases

**DOI:** 10.1186/s12931-022-02147-x

**Published:** 2022-09-19

**Authors:** Mellissa Gaudet, Maria Plesa, Andrea Mogas, Nour Jalaleddine, Qutayba Hamid, Saba Al Heialy

**Affiliations:** 1 Translational Research in Respiratory Diseases, Meakins-Christie Laboratories, Research Institute of the McGill University Health Center, Montréal, QC Canada; 2 College of Medicine, Mohammed Bin Rashid University of Medicine and Health Sciences, Dubai, United Arab Emirates; 3 College of Medicine, University of Sharjah, Sharjah, United Arab Emirates

**Keywords:** Vitamin D (1,25(OH)_2_D_3_), Airway inflammation, Airway infections, Respiratory diseases, Immunomodulator

## Abstract

Chronic airway inflammatory and infectious respiratory diseases are the most common medical respiratory conditions, associated with significant morbidity and mortality. Vitamin D (1,25(OH)_2_D_3_) deficiency has been shown to be highly prevalent in patients with chronic airway inflammatory and infectious diseases, correlated with increased disease severity. It has been established that vitamin D modulates ongoing abnormal immune responses in chronic respiratory diseases and is shown to restrict bacterial and viral colonization into the lungs. On the contrary, other studies revealed controversy findings regarding vitamin D efficacy in respiratory diseases. This review aims to update the current evidence regarding the role of vitamin D in airway inflammation and in various respiratory diseases. A comprehensive search of the last five years of literature was conducted using MEDLINE and non-MEDLINE PubMed databases, Ovid MEDLINE, SCOPUS-Elsevier, and data from in vitro and in vivo experiments, including clinical studies. This review highlights the importance of understanding the full range of implications that vitamin D may have on lung inflammation, infection, and disease severity in the context of chronic respiratory diseases.

## Introduction

Over the past several years, increasing interest has been attributed to the role of vitamin D deficiency in the severity of chronic respiratory diseases [1–3]. Several studies have provided evidence on how vitamin D deficiency/supplementation may alter the risk factors associated with the pathogenesis of chronic airway inflammatory and infectious respiratory diseases [4, 5]. The present paper will review the last 5 years of literature regarding the prevalence of vitamin D deficiency/supplementation and the prevention of chronic respiratory disease, which comprises allergic inflammation, chronic obstructive pulmonary disease (COPD), cystic fibrosis (CF), idiopathic pulmonary fibrosis (IPF), tuberculosis (TB), lung cancer, and the ongoing COVID-19 associated lung diseases. Using clinical, in vivo*,* and in vitro experimental studies, we will discuss proposed mechanisms of action of vitamin D.

## Vitamin D and asthma

It has been recently documented that a higher prevalence of vitamin D deficiency was reported in people suffering from chronic lung diseases [4] and asthmatics are considered at risk for vitamin D deficiency [4, 6]. Various studies have emphasized the association between low levels or deficiency of vitamin D with increased risk of asthma and other respiratory disease symptoms [7–10] including lower lung function [11]. This raises the question of whether low vitamin D is caused by disease severity or is a risk factor that can be easily remediated with supplementation [6].

Several studies involved clinical trials of vitamin D supplementation on asthma symptoms and control. Some have reported positive results while others have failed to do so. Nonetheless, secondary outcomes of these trials have led to interesting observations in certain subgroups. In a systematic review by Martineau et al*.*, they concluded that vitamin D reduces the risk of asthma exacerbations [12]. In another meta-analysis, it was also shown that vitamin D reduces the rate of asthma exacerbations requiring treatment with systemic corticosteroids. Interestingly, this study [13] and other studies have highlighted the benefit of vitamin D in individuals with low baseline levels of 25(OH)D [14, 15]. Wang et al*.*, for example, have shown that asthmatic patients with vitamin D insufficiency (vitamin D < 30 ng/ml) displayed reduced rate of asthma exacerbation by 27% upon vitamin D supplementation. Additionally, patients with lung dysfunction due to air limitation showed improvement upon vitamin D supplementation [16]. However, one study by Chen et al*.* showed that vitamin D did not improve asthma control test (ACT) score nor lung function among asthmatic patients treated with corticosteroids [17]. In another observation that was presented in a cross-sectional analysis, Rafiq et al*.* did not report any association between vitamin D concentrations and airway inflammation [18]. However, they did document high vitamin D levels to be associated with better lung function and reduced airway inflammation in obese individuals (body mass index; BMI ≥ 30 kg/m^2^) [18]. These observations would suggest that higher vitamin D levels would be more protective in overweight individuals. Despite the controversial data on vitamin D supplementation, supplementation could be beneficial in improving resistance to overall respiratory infections, particularly when administered on daily basis due to its immunomodulatory role [19, 20]. Mahboub et al. have shown that vitamin D supplementation (50,000 IU) in asthma patients with vitamin D deficiency (less than 20 ng/mL) improves steroid response by increasing the expression of glucocorticoid receptor (GR-α) and reducing blood levels of the asthma related cytokines IL-17F and IL-4 [21].

Vitamin D deficiency in children or early life has been associated with asthma development, where low vitamin D intake in mothers could influence wheezing in children [22]. Several mechanisms have been proposed to explain the preventive properties of vitamin D in children: (1) support tolerogenic immune responses (2) improve antiviral and antibacterial immune responses (3) enhance barrier properties of epidermis and (4) influence lung development [23]. Liu et al*.* showed a positive correlation between vitamin D and lung function in children [24]. Hornsby et al*.* suggested that neonatal immune responses could prevent the development of asthma [25]. Neonates from mothers supplemented with 4400 IU/d vitamin D_3_ had enhanced innate immune fitness [25]. Although there is accumulating evidence of the protective effects of vitamin D supplementation in neonates and in early life against asthma [26] it is still unclear what are the appropriate doses for optimal protection during pregnancy and early childhood. Controversially, it has been suggested that vitamin D supplementation might not have any protective effect in childhood asthma. Out of 18 RCTs, the pooled meta-analysis did not find any significant correlation between vitamin D supplementation on asthma attacks requiring rescue systemic corticosteroids. In addition, vitamin D did not reduce the severity nor the hospitalization rates of these patients [27]. Similarly, Nitzan et al*.* showed no supporting evidence that vitamin D supplementation in asthmatic children improves their asthma control and lung function [28]. However, in another meta-analysis and systematic review, the risk of 1200 after high-vitamin D supplementation was not increased in children aged from 0 to 6 years [29]. Interestingly, vitamin D supplementation was found to correlate with eosinophil counts and Immunoglobulin E (IgE) levels in children with asthma [30]. This coincides with a previous study where vitamin D supplementation was shown to reduce eosinophilic airway inflammation in patients with severe asthma [31].

**Fig. 1:**
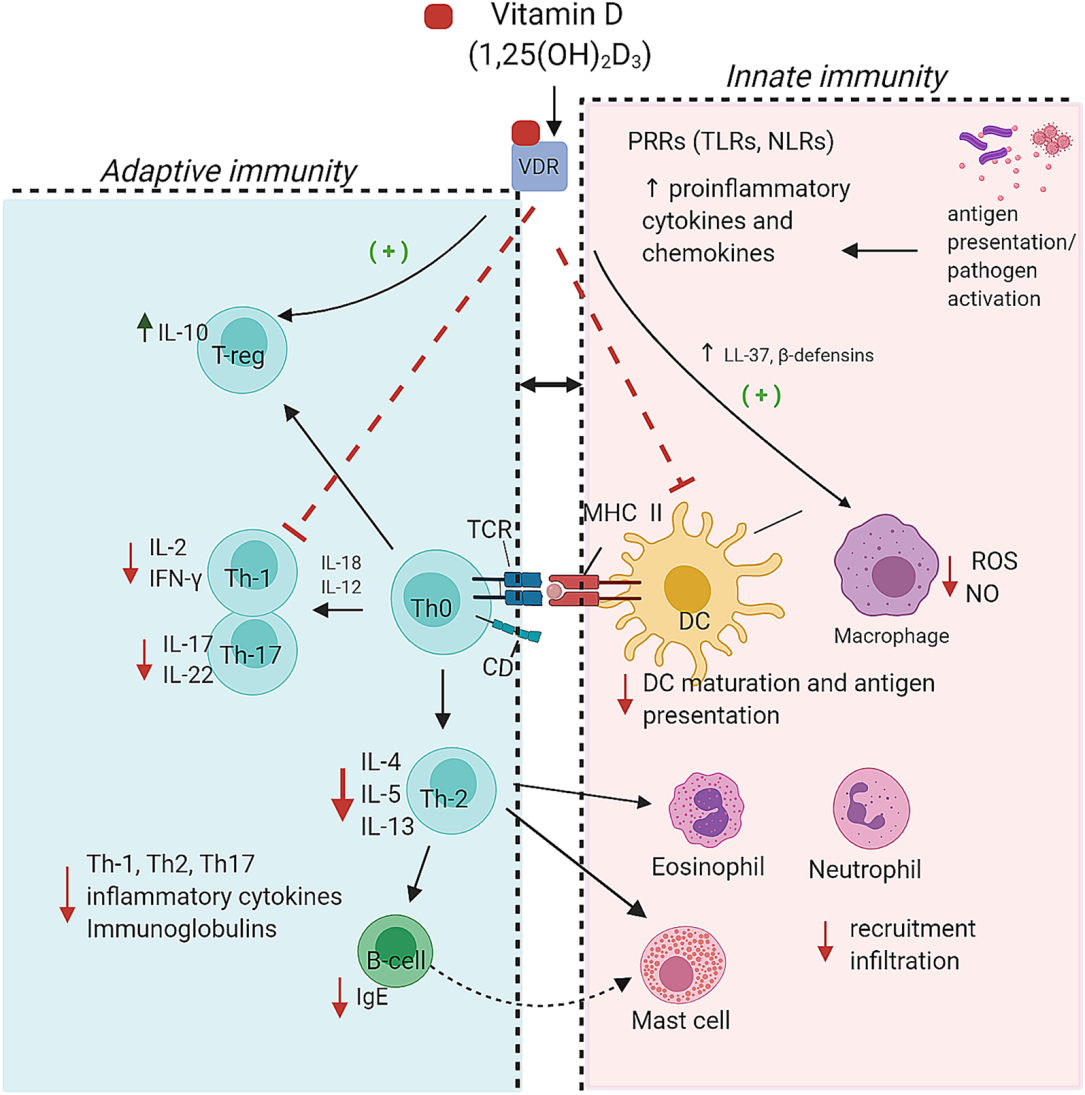
Summary of effects of vitamin D on innate and adaptive immune responses in the lung. Vitamin D has been shown to reduce inflammation and increase interleukin (IL)-10 levels. Vitamin D also modulates effector cells involved in the pathogenicity of allergic asthma: decreases mast cell activation, eosinophil count and infiltration of lung tissue. This inter play between innate and adaptive immune response emphases the potential of vitamin D to influence immune responses to allergens and build tolerance. Vitamin D has been shown to suppress the differentiation of Th1 cells and increase the production of anti-microbial peptides (LL-37 and β-defensins) by phagocytic cells. *VDR* Vitamin D Receptor, *PRRs* pathogen recognition receptors, *TLRs* Toll-like receptors, *NLR* nucleotide-binding oligomerization domain (NOD)-like receptors, *TCR* T-cell receptor, *DC* dendritic cells, *MHC II* major histocompatibility complex class II, *CD* co-stimulatory domain, *IL* interleukin, *Th-* T helper cells, *T-reg* regulatory T cells, *TNF-α* tumor necrosis factor alpha, *ROS* reactive oxygen species, *NO* nitric oxide, *IgE* Immunoglobulin E, * +* vitamin D, beneficial effect

**Fig. 2:**
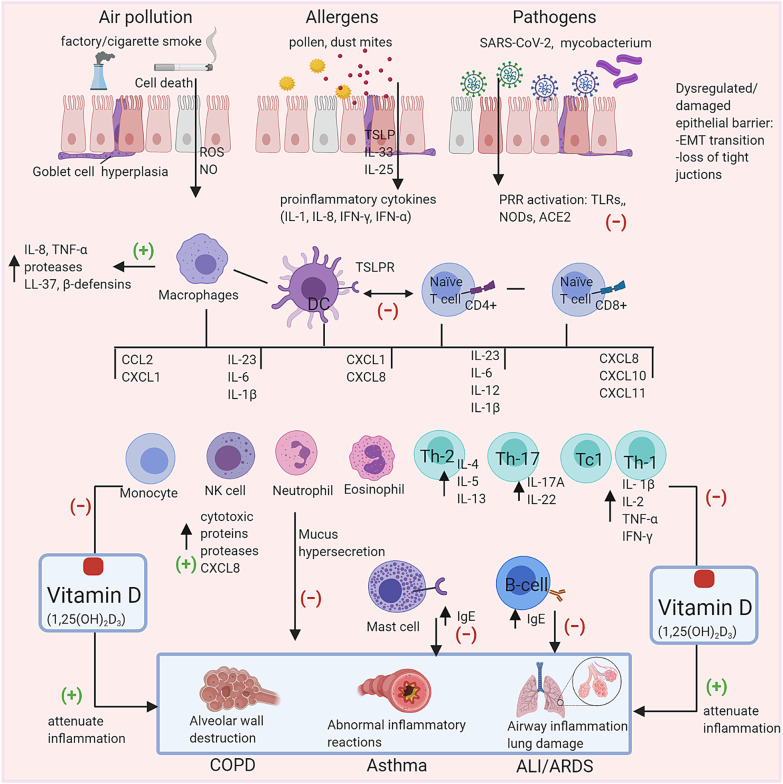
Overview of vitamin D on regulating inflammatory responses in respiratory diseases. Exposure to air pollutants, allergens or pathogens activates epithelial cells and alveolar macrophages releasing cytokines and chemokines. Inhaled smoke irritants increase the production of reactive oxygen species (ROS) and nitric oxide (NO) by epithelial cells. Alveolar macrophages and neutrophils release proteases to degrade the extracellular matrix (ECM) proteins resulting in alveolar wall destruction. Dendritic cells are the link between innate and adaptive immunity, involved in antigen recognition and phagocytosis. Vitamin D reduces the symptoms of caused by these irritants and pathogens. *PRRs* pathogen recognition receptors, *TLRs* Toll-like receptors, *NLR* nucleotide-binding oligomerization domain (NOD)-like receptors, *ACE2* angiotensin-converting enzyme 2, *TCR* T-cell receptor, *MHC II* major histocompatibility complex class II, *CD* co-stimulatory domain, *IL* interleukin, *Th-* T helper cells, *TNF-α* tumor necrosis factor alpha, *IFN* interferon, *IgE* immunoglobin E, *NK* natural killer cell, *EMT* Epithelial-mesenchymal transition, *COPD* chronic obstructive pulmonary disease, *ALI/ARDS* acute lung injury/acute respiratory distress syndrome, − /  + vitamin D, inhibitory/beneficial effect

Translational research has demonstrated considerable immunomodulatory effects of Vitamin D (Fig. 1). Many of these modulatory properties have been shown to positively influence the underlying mechanisms that contribute to asthmatic inflammation. On account of its anti-inflammatory properties, vitamin D has been shown to reduce inflammation [32, 33] and increase interleukin (IL)-10 levels [33] in respect to allergic asthma. This underlines the potential of vitamin D to influence immune responses to allergens and build tolerance. Vitamin D could also play a role in the effector cells involved in the pathogenicity of allergic asthma. It decreases mast cell activation [23], eosinophil count [34] and infiltration of lung tissue [33]. Moreover, it has been shown to decrease phospholipase A_2_ production, an important contributor to asthma pathogenesis. In this context, phospholipase A_2_ is involved in the degradation of surfactants and in part is responsible for the release of cytokines [35]. B cells have also been shown to be affected by vitamin D, more importantly by inhibiting their proliferation and differentiation to plasma cells [32] (Fig. 2).

Vitamin D actions on inflammatory mechanisms has also been featured in non-allergic asthma. Extensive research has been done on the underlying processes involved in airway tissue remodeling in asthma, specifically on structural cells implicated in this irreversible process. Pfeffer et al. showed that vitamin D decreased oxidative stress in primary human bronchial epithelial cells (HBEC) and suppressed IL-6 response from both healthy and asthmatic donors. Of note, the airway epithelium is an important barrier and regulator of inflammation and immune responses in this process [36]. Vitamin D has been shown to reduce proliferation, proinflammatory cytokines, matrix metalloproteinase (MMP) in airway smooth muscle cells [32], factors contributing to airway inflammation and remodeling. In another study, it was shown that vitamin D in airway smooth muscle cells attenuates TLR3 agonist-induced inflammatory and fibrotic responses by a viral antigen [37].

Among severe asthmatics, a subset of these patients is said to have steroid-resistant asthma, a phenotype linked to IL-17. Several studies have focused on the implication of T cells in asthmatics with this severe phenotype. Vitamin D was shown to shift Th17 effector cells, important source of IL-17 and pro-inflammatory mediators, towards a Treg phenotype [32]. This regulatory shift is also denoted by a decrease in IL-17 production and a rise in IL-10 levels [32, 38, 39], thus possessing a protective outcome. Also, Sun et al*.* recently demonstrated that vitamin D inhibits the translocation of P65, a key transcription factor, involved in the cellular differentiation of CD4 + T helper cells into the Th17 lineage [38]. Neutrophil influx or neutrophilic inflammation in severe asthmatics is also thought to be caused in part by IL-17 [23]. Vitamin D was shown to reduce pro-inflammatory cytokine production in stimulated neutrophils and enhance antibacterial activity with reactive oxygen species (ROS) reduction [23], which would otherwise aggravate the inflammatory environment. These findings have been summarized in Fig. 2.

## Vitamin D and chronic obstructive pulmonary disease (COPD)

Among the published clinical studies, a hospital-based cross-sectional study reported that increased TNF-α level and low serum vitamin D concentrations are associated with airway obstruction in COPD patients. These results were predictable, knowing that TNF-α plays essential roles in pathogenesis and inflammatory responses in COPD, and that vitamin D has a modulator effect on TNF-α [40].

Another study investigated the correlation of serum 25(OH)D levels with COPD characteristics (lung function, exacerbations’ frequency and medication), despite a thorough adjustment for COPD confounders, the authors only found a significant effect of deficient 25(OH)D levels in the change of lung function parameters FEV_1_ and FVC [41].

On the other hand, a certain unpredictability and variability due to study design were also observed among clinical studies. For example, in two cross-sectional studies [42, 43], their primary objective was to investigate whether vitamin D deficiency can directly be associated with improved lung function/reduced exacerbations. Although both studies reported that COPD patients hospitalized for exacerbations are a risk group for vitamin D deficiency, both authors acknowledged some study limitations, as such: no possible comparison of hospitalized COPD with non-hospitalized COPD patients and that in elderly patients due to the multiple complications, vitamin D level and function would be affected.

Once more, the drawback of finding association of vitamin D supplementation in COPD-patients has been also observed in some RCT studies. In these studies [44, 45], vitamin D supplementation failed to show an overall effect on lung function (FVE_1_ or FVC) [45], nor an association with lower risk of exacerbations due to respiratory infections in vitamin D-deficient COPD patients [44]. In a recent study by Rafiq et al*.*, the effects of vitamin D supplementation in COPD patients with vitamin D deficiency was investigated. Their findings failed to show any preventive or positive effects of vitamin D supplementation on lung exacerbations [46]. They mentioned several factors contributing to their results. As compared to other studies where vitamin D reduced exacerbations in COPD patients with vitamin D deficiency [44, 47], the participants in this study displayed more severe COPD symptoms. Also, the study participants had a higher mean FEV1 at baseline, and the exacerbation rate was higher than that study done by Martineau et al*.* [44]. They also highlighted that the absence of an effect from vitamin D supplementation in COPD patients may be related to the fact that other contributory mechanisms such as inactivity and the chronic inflammatory state may override potential small negative effects of vitamin D deficiency on muscle strength [48–50]. Collectively, they concluded no effect of vitamin D supplementation on reducing exacerbations in COPD patients with vitamin D supplementation. Nonetheless, they highlighted the need of supplementing COPD patients with a serum 25(OH)D concentration < 124.8 ng/ml, as COPD patients having low vitamin D are diagnosed with osteoporosis and bone fractures [46, 47, 51, 52]. By contrast, other RCT studies that aimed similar clinical outcomes [53, 54], showed that vitamin D supplementation was associated with a decrease in COPD exacerbations, increase in FEV_1_ [53] and increase in serum 25(OH)D levels [54]. Overall, this highlights the notion that vitamin D deficiency is correlated with poor prognosis in COPD patients, and supplementation could possibly ameliorate disease severity. Numerous experimental [55, 56], and randomized clinical studies (RCT) [41, 42, 44, 45, 53, 54] have been developed over the past years, and have brought valuable data on the causal relationship between vitamin D deficiency/supplementation and COPD. Likewise, in a murine experimental study, Heulens et al*.* performed a comprehensive investigation on the effect of vitamin D deficiency and cigarette smoke exposure (CSE) on the development of COPD-like characteristics. They have shown that vitamin D deficiency is associated with decreased lung function upon CSE and contribute to early signs of emphysema [55]. Similarly, Cielen et al*.* investigated the involvement of vitamin D deficiency on skeletal muscle dysfunction in smoke-/air-exposed mice. They showed that vitamin D deficiency is associated with increased lung inflammation, while the highest emphysema score was only observed after prolonged exposure to smoke [56].

## Vitamin D and pulmonary fibrosis

### Cystic fibrosis

Many have highlighted the complexity and the challenges of cystic fibrosis (CF) regarding vitamin D. It is estimated that around 40–90% of CF patients are vitamin D deficient, with vitamin D serum levels below 30 ng/ml [57]. The CF lung environment may influence how vitamin D is metabolized because of the cystic fibrosis transmembrane regulator (CFTR) mutation in bronchial cells, which decreases its ability to activate vitamin D locally, leading to impaired antibacterial activity due to chronic inflammation. Through vitamin D treatment, levels of IL-17 present in the sputum of CF patients have been reduced [58]. Association has been made between impaired inflammatory responses in the airways, increased infection and decreased lung function with vitamin D deficiency [22], these effects are exacerbated with disease progression [58, 59]. Vitamin D potentially inhibits the activity of antigen presenting cells, the expression of inflammatory cytokines, and the differentiation of T lymphocytes. Therefore, vitamin D, through its anti-microbial and anti-inflammatory properties, is considered an adjuvant therapy in CF patients [60].

Promising results have come from clinical trials, demonstrating the positive effects of vitamin D supplementation in CF patients, namely in the potential of reducing inflammation, improving lung function [61, 62] and decreasing pulmonary exacerbations [60]. It was shown that higher 25(OH)D levels in CF patients are associated with reduced pulmonary exacerbations and better lung function with improved FEV1 [63]. In a study by Abu-Fraiha et al., it was suggested that CF patients receiving high doses of vitamin D displayed reduced pulmonary exacerbations [60]. This is explained by the fact that the epithelial layer of the airways, expressing vitamin D receptor (VDR), could convert inactive 25(OH)D to active 1,25(OH)2D [64, 65], however such conversion is found to be impaired in CF patients [60, 66]. Of note, this active form of vitamin D is known to exert its autocrine and paracrine functions, upregulating vitamin D correlated genes with important consequences in the local immunity of lungs by imposing its anti-inflammatory and antimicrobial properties [64, 66, 67]. This further highlights the correlation between vitamin D deficiency and respiratory viral infections.

Several other studies highlighted the involvement of vitamin D on the different immunological mechanism important in CF. Through vitamin D treatment, levels of IL-17 present in the sputum of CF patients have been reduced [59]. These results have been supported in a recent pilot study that measured a decrease of IL-17A and IL-23 levels in the exhaled breath concentrate of CF patients supplemented with 1,25(OH)_2_D_3_ and cholecalciferol [68]. Also, a decrease in IL-8 levels, an important neutrophil chemokine, by vitamin D has been the outcome of many studies [59, 69].

**Fig. 3:**
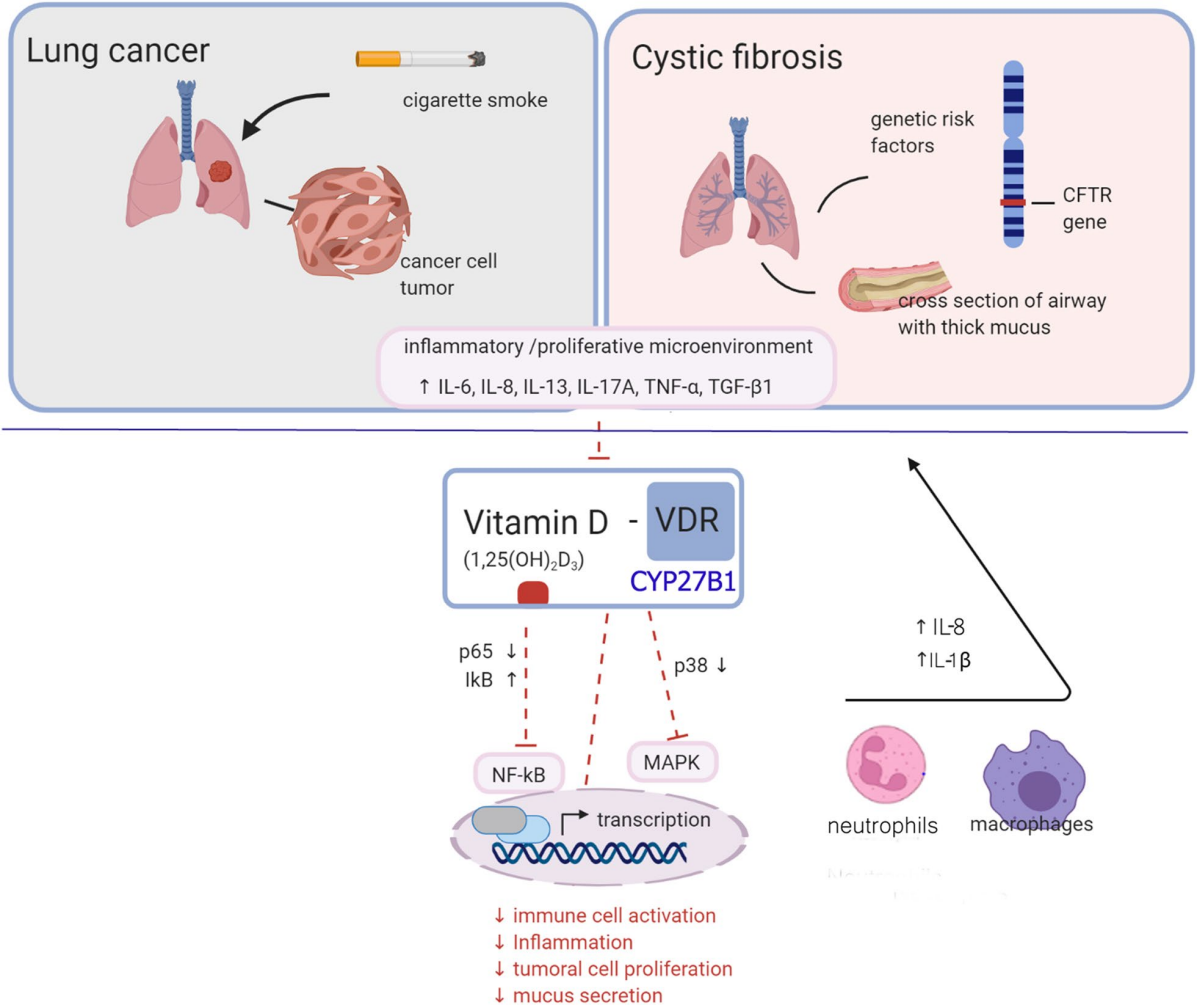
Overview of vitamin D in regulating inflammatory responses in lung cancer and cystic fibrosis. Tumor or cystic fibrosis inflammatory/proliferative microenvironment induces the expression of inflammatory cytokines, chemokines and growth factors which accelerate tumor growth, invasion, and mucus secretion. Vitamin D affects the inflammatory microenvironment inhibiting inflammatory and cell proliferation signaling pathways (NF-kB/MAPK). *NF-kB* nuclear factor-kappa B, *MAPK* mitogen-activated protein kinase, *CTL* cytotoxic T lymphocytes, *MHC II* major histocompatibility complex class II, *CFTR* cystic fibrosis transmembrane conductance regulator, *IL-* interleukin, *CD* co-stimulatory domain, *DC* dendritic cell, *IgE* immunoglobulin E, *TNF-a* tumor necrosis factor alpha,  + vitamin D, beneficial effect

Molecular mechanisms have also been proposed, supporting the anti-inflammatory actions of vitamin D to be through the regulation of p38 MAPK activity and the nuclear translocation of NF-κB [70], as summarized in Fig. 3. Although this study was conducted using intestinal epithelial knockdown cells and *Cftr *^*−/−*^ female mice, their data would suggest that a threshold of inflammation needs to be reached for vitamin D to be most effective [70].

Lung infection is a major contributor of airway inflammation in CF patients and in turn worsens disease severity. An innovative in vitro study demonstrated the protective effect of 25(OH)D against *Pseudomonas aeruginosa* infection when vitamin D is administered in the form of liposomes using nebulisation [71]. Vitamin D in liposomes was deemed more effective towards bacterial killing compared to simply being in solution [71]. A study by Chen et al*.* reported that neutrophils and macrophages treated with 1,25(OH)_2_D_3_ produced higher levels of IL-8 and IL-1β in the presence of bacterial stimuli compared to cells without vitamin D [72]. Leading them to propose that this increase in IL-8 production by neutrophils could enhance their ability to respond to pathogens, namely by recruiting more neutrophils [72]. Nonetheless, they also reported that bronchial lung epithelial cells treated with vitamin D produced more IL-8 after LPS stimulation compared to cells not treated with vitamin D, in addition to reducing phagocytosis in macrophages [72]. The results from Chen et al*.* would suggest that the underlying mechanism of vitamin D on responses to infection is more complex and would be cell dependent and dependent on pre-existing inflammation [72].

In accord with other research, the effector cells involved in CF inflammation are also affected by vitamin D. The study of Pincikova et al*.* suggested that vitamin D reduces the amount of co-stimulatory molecule CD86 on myeloid dendritic cells [73]. They also showed that CD8 + T cell activation and exhaustion as well as CD4 + T cells were impacted upon vitamin D treatment in CF patients [73]. The neutrophil counts were also shown to be negatively correlated with free serum 25(OH)D levels in CF patients supplemented with vitamin D [61].

Finding new therapeutic avenues for CF is imperative and with the development of modulators and potentiators of the CFTR protein, the status of some CF patients have improved. However, these drugs are only suited to correct a subgroup of CFTR mutations, limiting their benefits to only certain patients. DiFranco et al*.* showed that 1,25(OH)_2_D_3_ increases both mRNA and protein CFTR levels in both normal and CF cells. They also showed that topical administration of vitamin D to cells and mice was able to confer the same outcome [74]. These are important findings for the development of new therapies for CF patients, in addition to having the potential for improving the CFTR correctors already available.

### Idiopathic pulmonary fibrosis (IPF)

Idiopathic pulmonary fibrosis (IPF) is one of the aggressive forms of interstitial lung fibrotic diseases, characterized by activated fibroblasts and extracellular matrix (ECM) degradation [75, 76]; as seen in different chronic lung diseases. Damages targeting ageing alveolar epithelium is thought to play a key role in this process, due to the secretion of pro-fibrotic mediators and the activation of mesenchymal cells that results in an irreversible form of premature and senescence of epithelial cells [77]. Similar to the previously mentioned lung diseases, vitamin D deficiency have also been shown to negatively impact IPF patients, correlating with enhanced disease severity and a higher mortality rates [78]. This was proven by Tzilas et al*.* using a bleomycin induced pulmonary fibrosis mouse model. Interestingly, treatment with vitamin D reduced the responsiveness of lung fibroblasts to pro-fibrotic stimuli and significantly decreased ECM markers. These changes were associated with restoration of the vitamin D receptor (VDR), after it’s downregulation via TGFβ1-Smad3 phosphorylation pathway. Concluding vitamin D being a promising prognostic and potential therapeutic agent in these patients [78], however, further studies are sorely needed.

## Vitamin D and tuberculosis

Pulmonary tuberculosis (TB), caused by the Mycobacterium tuberculosis (Mtb), is a global health problem being the leading cause of death. The World Health Organization (WHO) estimates that there are over 10.4 million new individuals with pulmonary TB every year worldwide [79]. Several animal and clinical studies aimed to investigate the potential benefit in using vitamin D supplements as an adjuvant to a standard TB therapy to improve the clinical parameters. Some observational studies have established a potential benefit of vitamin D supplementation as an adjuvant treatment to ameliorate TB severity [80, 81]. Although, RCTs and experimental animal studies showed limited effects on Mtb clearance and infection outcomes [82–84]. In the last five years, several RCT studies have been performed, where oral vitamin D supplements alone [82, 83, 85, 86] or in combination with antimicrobial agents, such as 4-phenyl butyrate (PBA) [84] have been used as potent inducers of host defenses against Mtb infections. Interestingly, most RCT studies confirmed high rates of vitamin D deficiency in patients with pulmonary TB and that adjunctive doses of vitamin D led to a substantial increase in plasma 25(OH)D concentrations [82, 86], with no enhanced sputum Mtb clearance. This is possibly due to the significant difference in the time of the conversion of a positive Mtb sputum culture to negative [82, 83, 85] upon vitamin D administration compared with a placebo (p > 0.05). In a study by Jolliffe et al*.*, individual participant data (IPD) from the meta-analysis of 1850 participants from 8 studies could identify factors explaining this variation. They meta-analyzed IPD from RCTs of vitamin D in patients receiving antimicrobial therapy for pulmonary TB. Primary and secondary outcomes related time-to sputum culture conversion and time to sputum smear conversion were assessed. It was found that vitamin D did not influence time to sputum culture conversion overall, but it accelerated sputum culture conversion in patients with multidrug-resistant pulmonary TB. It also accelerated time to sputum smear conversion overall [87]. In another placebo-controlled randomized study [86], intramuscular vitamin D added to TB therapy substantially improved rates of sputum smear conversion in patients of pulmonary TB, although it also had some limitations. Among them, the study was not double-blinded, and placebo was not given to the group randomized for the anti-tuberculosis therapy only, which potentially introduced risk of “false positive” and the difference observed between groups could possibly be attributed to the psychological effect of intramuscular injection of vitamin D in the intervention group. A combined adjunct therapy of vitamin D supplements and PBA [84] has been shown to increase sputum culture conversion in week 4 compared to vitamin D supplements alone or to the placebo group. Additionally, combined adjunct treatment vitamin D-PBA also resulted in an increased expression of LL-37 in alveolar macrophages, along with an enhanced intracellular killing of Mtb in macrophages, ex vivo. These findings suggest that there was a synergistic effect between vitamin D and PBA. Overall, RCTs published data on the impact of adjunctive vitamin D administration in patients with pulmonary TB are difficult to interpret due to broad variability among them, including the readout time point for culture conversion of 4 weeks [84] versus 8 weeks [82, 83]; randomization of TB patients' treatment-naïve [85, 86] versus TB patients that previously received a TB therapy or with reactivation of the TB disease; uncertain randomization assignment [86], variability among vitamin D doses, types of administration and dosing schedules [82, 83, 85, 86] or the absence of measured serum 25(OH)D concentrations at baseline [83, 85]. Additionally, all studies presented low number of subjects in each randomized treatment arm and experienced high withdrawal rates (24%) or missing data for analysis, which may contribute to the poor clinical outcomes of vitamin D supplementation as an adjuvant treatment for TB infection [82, 83, 85, 86, 88].

As provided by in vivo studies, 1,25(OH)_2_D_3_ ameliorates TB disease severity. It has been shown that 1,25(OH)_2_D_3_ suppress the differentiation of Th1 cells, which are usually involved in exacerbated immune responses against TB disease, and it favors the differentiation of anti-inflammatory regulatory T cells. Additionally, it has been reported that 1,25(OH)_2_D_3_ also affects Mtb survival, intracellularly, through increased production of antimicrobial peptides (LL-37 and β-defensins) by phagocytic cells [84] (Fig. 1). These 1,25(OH)_2_D_3_ antimicrobial actions could not be validated in rodent macrophages due to the absence of VDRE regulatory DNA sequences in these genes. Nonetheless, in vitro data suggests that 1,25(OH)_2_D_3_ increases the host defense cellular mechanisms against Mtb infection, possibly leading to better antimicrobial action against Mtb [89].

In murine models, 1,25(OH)_2_D_3_ treatment altered the granulomatous response to Mtb infection and decreased B cell lymphocytic aggregates. Additionally, a significantly higher bacterial burden was found in the lungs of 1,25(OH)_2_D_3_-treated mice compared to the control group, hence implying the potential benefit of vitamin D supplementation in a better clearance of Mtb infection [89].

## Vitamin D and lung cancer

Lung cancer, one of the most common cancers worldwide [90], is associated with a poor prognosis and is a leading cause of high mortality rate in both men and women. More than 85% of lung cancer cases are classified as non-small cell lung cancer (NSCLC) with a predicted survival rate of 15% [91]. Tobacco smoke is the most common etiology for lung cancer and accounts for approximately 85% of lung cancer deaths in the United States of America (USA) alone [92]. Despite the overall decrease of lung cancer incidence rates due to the decreased rate of smoking, the rates of women and former or never smokers are still increasing [93–95].

Vitamin D has been extensively studied in different types of cancer; in vitro and in vivo. In vitro studies of lung cancer, in addition to other types of cancers [96, 97], demonstrated vitamin D to have an anti-metastatic role, by inhibiting cell proliferation and decreasing invasiveness [95, 97]. Moreover, studies indicate that vitamin D status correlates with overall lung cancer survival. In the Lewis lung carcinoma animal model, vitamin D supplementation prolonged the overall survival (OS) with evidence of anti-metastatic effects [95]. As for studies involving in vivo, vitamin D is associated with improved survival of early-stage non-small cell lung cancer (NSCLC), a phenotypic subtype of lung cancer, as patients who had higher intake of vitamin D, as demonstrated by higher serum levels of 25(OH)D, had a better prognosis than patients with reduced 25(OH)D serum levels [5, 98]. Surprisingly, it is evidenced that serum vitamin D level was not directly correlated with the survival of patients and rather the observed vitamin D receptor (VDR) polymorphisms are strongly associated with worse outcomes [99]. Findings were further confirmed in a randomized, double-blinded, placebo-controlled trial to determine whether vitamin D supplementation improved overall survival (OS) or relapse-free survival (RFS) in the total study population or in relation to stage, pathology, or serum levels of 25(OH)D before vitamin D supplementation of 1200 IU for 12 months. Supplementation did not improve OS in the total studied population. However, there was a statistically significant increase in OS and RFS in the subgroup of patients with early-stage NSCLC [100].

In another meta-analysis on the relationship between vitamin D and lung cancer risk and outcomes, 22 studies were analyzed. The data suggested that vitamin D intake and high serum 25(OH)D level correlated with better prognosis, lower lung cancer overall risk and increased survival. Interestingly, detected levels of vitamin D were higher in nonsmokers when compared to smokers, and this correlated with decreased lung cancer risk [101]. It is important to note that extensive research has also involved assessing VDR. Studies showed a negative association between VDR and CYP27B1 expression and correlated it with lung cancer survival where the pro-inflammatory cytokines, IL-6 and TNF-α are involved in the downregulation of these genes (**Fig. ****3****)**. Nonetheless, in recent studies, it has been shown that treatment such as Nivolumab, a monoclonal antibody which binds to anti-programmed cell death protein 1 (PD-1) can be affected by vitamin D concentrations prompting the suggestion to evaluate vitamin D levels prior to treatment [102].

## Acute lung injury/acute respiratory distress syndrome and COVID-19

Acute lung injury (ALI) and Acute respiratory distress syndrome (ARDS) remain a significant cause of morbidity and mortality in critically ill patients worldwide. Pathologically, it is characterized by an influx of immune cells, notably macrophages and neutrophils, leading to endothelial and epithelial damage, and thus overall alveolar damage [103]. To date, no known therapy has proven to be beneficial in ALI/ARDS patients. Recent evidence shows an association between vitamin D deficiency and increased adverse outcomes in patients who develop ALI/ARDS. In a study by Dancer et al*.* it was shown that vitamin D deficiency was common among ARDS patients [104]. Moreover, in a mouse model of ARDS, dietary-induced vitamin D deficiency resulted in hypoxia, epithelial damage, and alveolar inflammation. This observation was further strengthened in the clinical setting where vitamin D-deficient patients were supplemented with vitamin D prior to oesophagectomy which resulted in reduced alveolar capillary damage [105, 106]. Using in vivo investigations, intragastric vitamin D supplementation prior to intratracheal LPS administration to deficient mice led to an increase in alveolar epithelial type II cell proliferation, decrease in apoptosis and decrease in epithelial mesenchymal transition (EMT). In vitro, it was found that alveolar epithelial type II (ATII) proliferation was increased through the PI3K/Akt signaling pathway and EMT via TGF-β was reduced [107].

Interestingly, the findings associating vitamin D and ALI/ARDS has led researchers to study its effects in COVID-19, which involves the development of ARDS symptoms. The 2019 novel coronavirus (COVID-19), caused by SARS-CoV-2, is a pandemic with high morbidity and mortality rates. In most patients, the symptoms have been described as mild to moderate with dry cough, fever, and shortness of breath. However, in some patients symptoms are classified as severe and, in some situations, critical [108, 109]. SARS-CoV-2 has been shown to bind to angiotensin converting enzyme 2 (ACE2) present on a multitude of cells and more notably on respiratory epithelial cells [110]. A significant number of COVID-19 patients have been shown to exhibit ARDS as a major complication due to the induction of different mechanism that involves cytokine storm and dysregulation of the ACE system [111–113]. Moreover, recent evidence points to another driver of the disease: vitamin D deficiency [113, 114]. A 2020 study found a negative correlation between vitamin D levels and COVID-19 cases and deaths in 20 different European countries [115]. In another study, patients with COVID-19 in the intensive therapy unit (ITU) showed higher rates of vitamin D deficiency compared to patients who were not admitted to ITU [116]. Additionally, the authors have proposed that the mechanisms through which vitamin D deficiency may lead to worse outcomes in COVID-19 is through the suppression of antiviral peptides such as LL37 which has various immunomodulatory roles [117]. It is suggested that ARDS in COVID-19 patients could be reduced by activating the vitamin D receptor, endorsed by calcifediol (25OHD) supplementation [113]. Vitamin D deficiency has been shown to cause over-activation of the dual RAS system of which ACE2 is an important regulator [118]. It has also been proposed that vitamin D can inhibit the ACE/Ang II/AT1R axis of the RAS pathway [118, 119], providing protection against ARDS. Due to the many immunomodulatory effects of vitamin D, researchers worldwide are interested to see the effect of vitamin D as a potential therapeutic target in COVID-19. In a recent study (SHADE study), forty SARS-CoV-2 positive patients with vitamin D deficiency were randomized to receive daily cholecalciferol for 7 days or placebo. In response to supplementation, a greater number of these individuals who received cholecalciferol turned SARS-CoV-2 RNA negative and showed a significant decrease in fibrinogen [120]. However, to date, research is still ongoing and the role of vitamin D in COVID-19 remains to be verified.

## Vitamin D therapy: future directions

Research involving vitamin D optimization, combinational therapies and novel technologies is growing. Further exploration of the different vitamin D metabolites and analogs and their effects are needed. The question of dosage, duration, and age to start administering vitamin D are also a reoccurring preoccupation in the literature, mainly because of inconsistencies between studies. Promising work is being conducted on new methods of administration of vitamin D at target sites, namely novel drug delivery systems using nanoparticles. This technology showed promising results in the treatment of different lung diseases (asthma, COPD, lung cancer and TB) [121–123], although only few studies have demonstrated its potential as a method to administer vitamin D directly in the lungs.

A growing interest in the microbiota has led to the investigation of different sources of inflammation and dysregulation of immune responses influenced by the lung microbiota [124] as well as the “gut-lung axis” of the microbiota [125]. In turn, questions on metabolism were raised and a recent study showed that asthma and COPD patients have a reduced response to vitamin D supplementation due to dysregulated vitamin D metabolism [126], which is also the case in CF [88]. This highlights the need for new methods of improving vitamin D absorption and bioactivity. Such as, the encapsulation of vitamin D in nanomaterials, shown to enhance these features, broadening the efficacy of vitamin D in fortified foods and supplements [127]. New combination therapies are emerging, introducing effective candidates that combine vitamin D and corticosteroids for the treatment of asthma. One of these being from Choi et al*.* who developed a dexamethasone-conjugated moiety combined with an anti-vitamin D binding protein siRNA formed into nanoparticles, which significantly reduced airway inflammation and other asthma-induced responses in ovalbumin sensitized mice [128]. Other studies have also suggested a synergistic effect in reducing airway inflammation when combining glucocorticoids and vitamin D [129–131]. Preclinical studies have also reported an antitumor effect due to 1,25(OH)_2_D_3_ and dexamethasone combined therapy; in vivo mouse models [132]. As for COVID-19 therapeutic approaches, using combinational treatment with vitamin D should also be considered in future investigations. It has been evident that probiotics are considered preventive treatment in COVID-19, reducing severity of infection [133]. Together with vitamin D, such combinational treatment could potentially be an important player in modulating the exaggerated immune responses in SARS-CoV2 infected patients, leading to better clinical outcomes.

## Conclusion

In this review, we highlight the importance of vitamin D in the inflammatory mechanisms underlying the different respiratory diseases presented. Vitamin D has emerged as a potential therapeutic target in patients with chronic and acute airway diseases, including patients of COVID-19. However, controversy in the available data still remains where some studies showed as provided by in vitro studies more beneficial outcomes than others, potentially explaining why we still do not have an explicit therapeutic foundation It is important to note that the therapeutic considerations for vitamin D are increasing due to its anti-inflammatory and anti-oxidative effects despite the controversial data. Vitamin D supplementation could be beneficial in improving overall outcomes in various respiratory diseases, particularly when administered on daily basis and when administered as an adjunctive therapy.

## Data Availability

Not applicable.

## Abbreviations

ALI: Acute lung injury
ACE: Angiotensin converting enzyme
ARDS: Acute respiratory distress syndrome
COPD: Chronic obstructive pulmonary disease
CF: Cystic fibrosis
CFTR: Cystic fibrosis transmembrane regulator
CHD: Coronary heart disease
IL: Interleukins
IPF: Idiopathic pulmonary fibrosis
MMP: Matrix metalloproteinase
MAPK: Mitogen-activated protein kinase
NF-kB: Nuclear factor-kappa B
ROS: Reactive oxygen species
RCT: Randomized clinical studies
SARS-CoV-2: Severe acute respiratory syndrome- corona virus-2
TB: Tuberculosis
TGF-β: Transforming growth factor-beta
TNF-α: Tumor necrosis factor-alpha
Th: T-Helper cells
TLRs: Toll like receptors
